# Comparison of outcomes of extracorporeal shock wave lithotripsy versus retrograde intrarenal surgery in the management of renal calculi measuring one to two centimeters

**DOI:** 10.12669/pjms.42.9.16588

**Published:** 2026-09

**Authors:** Muhammad Waqas Arshad, Atif Javed, Shafeullah Ahadi, Azhar Ghayoor Yousaf

**Affiliations:** 1 Dr. Muhammad Waqas Arshad, MBBS. Department of Urology, Ziauddin Hospital, Karachi, Pakistan; 2 Dr. Atif Javed, FCPS, MBBS. Department of Urology, Ziauddin Hospital, Karachi, Pakistan; 3 Dr. Shafeullah Ahadi, MBBS. Department of Urology, Ziauddin Hospital, Karachi, Pakistan; 4 Dr. Azhar Ghayoor Yousaf, MBBS. Department of Urology, Ziauddin Hospital, Karachi, Pakistan

**Keywords:** Extracorporeal shock wave lithotripsy, Retrograde intrarenal surgery, Renal stones, Stone clearance, Retreatment

## Abstract

**Objectives:**

To compare outcomes of extracorporeal shock wave lithotripsy (ESWL) and retrograde intrarenal surgery (RIRS) for renal stones measuring 1-2 cm, including stone clearance, hospital stay, hematuria, sepsis, postoperative pain, and retreatment.

**Methodology:**

Six months, single-center, open-label, parallel-group randomized controlled trial was conducted at the Department of Urology, Dr. Ziauddin Hospital, North Nazimabad, Karachi, from September 5, 2025 to March 4, 2026. Ninety-eight eligible adults were enrolled consecutively and randomized 1:1 to ESWL or RIRS by lottery method using concealed identical allocation slips. Non-contrast CT-KUB was performed at baseline and at 28 days. Pain was assessed by VAS at 24 hours. Data were analyzed using SPSS version 26, with p<0.05 considered significant.

**Results:**

Baseline characteristics were comparable between the treatment groups. Stone-free status after one session was 53.1% with ESWL and 85.7% with RIRS (RR 1.62, 95% CI 1.21 to 2.15; p<0.001), while 28-day stone clearance was 67.3% and 85.7%, respectively (RR 1.27, 95% CI 1.02 to 1.60; p=0.032). ESWL was associated with shorter hospital stay (p<0.001). Median VAS pain score at 24 hours was higher after ESWL [4 (IQR 3-4)] than RIRS [3 (IQR 2-4); p<0.001]. Retreatment was more frequent after ESWL (30.6% vs 10.2%; RR for RIRS vs ESWL 0.33, 95% CI 0.13 to 0.85; p=0.012), while gross hematuria and postoperative sepsis were comparable.

**Conclusion:**

RIRS provided higher clearance and lower retreatment and pain scores than ESWL, while ESWL was associated with shorter hospital stay.

***Registration Number:*** www. clinicaltrials.gov (NCT07261995).

## INTRODUCTION

Renal stones affect approximately 1% to 13% of the global population varies across different region, with higher prevalence in regions known as the “stone belt,” including parts of Asia, the Middle East, and Western countries. In Pakistan, renal stone disease is highly prevalent, particularly among males and individuals aged 21–45 years.^1^,^2^ Calcium oxalate stones constitute around 80% of cases, followed by uric acid stones. Key risk factors include hot climates, high intake of animal protein, low fluid consumption, obesity, and metabolic syndrome.^3^ Rural populations and individuals from lower socioeconomic backgrounds are disproportionately affected. Regional differences exist, with northern areas reporting more oxalate stones. Poor hydration, dietary imbalances, and sedentary lifestyles remain the major contributors to stone formation in the Pakistani population.^4^,^5^

**Fig.1:**
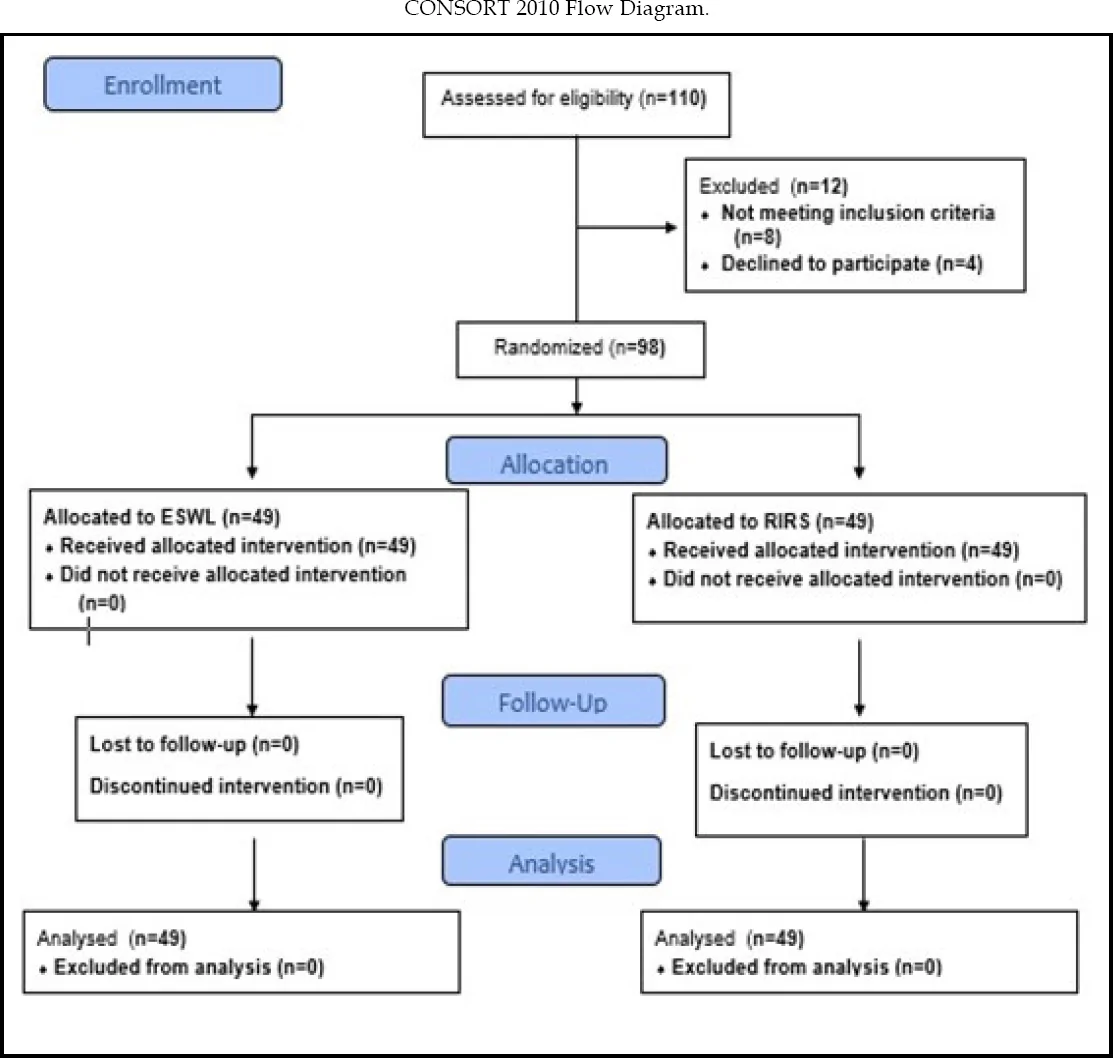
The completed standard CONSORT 2010 flow diagram is provided at the end of the manuscript.

Extracorporeal shock wave lithotripsy (ESWL), retrograde intrarenal surgery (RIRS), and, in certain patients, mini-percutaneous nephrolithotomy (Mini-PCNL) are frequent therapeutic options for renal stones that measure 1-2 cm. RIRS often has better clearance rates (85–87%) compared to ESWL (63–77%) and usually needs fewer follow-up procedures.^6^–^8^ RIRS usually requires anesthesia and carries procedural risks like ureteral injury, whereas ESWL is non-invasive, performed without anesthesia, and generally safer, but usually requires multiple sessions, especially for lower-pole stones.^7^ Cost and surgeons’ experience also influence the choice, with RIRS being a costly procedure and demanding higher surgical expertise. Patient factors, stone location, and resource availability can affect the treatment selection.^9^–^11^

Although ESWL and RIRS are recognized treatment options for 1-2 cm renal stones, uncertainty remains about their comparative performance when 28 days CT-confirmed clearance, early pain, retreatment, hospital stay, and short-term adverse events are considered in the same clinical population. Available comparative studies are frequently restricted to lower-pole stones, selected subgroups, or non-randomized designs, and a well-structured randomized control trial from Pakistan remains limited. This gap is clinically relevant because stone characteristics, patient profiles, access to urologic facilities, and retreatment burden may influence treatment choice. The study was undertaken to determine whether ESWL and RIRS differ in clinically relevant outcomes among adults with a single 1-2 cm renal calculus. The primary objective was to compare 28 days stone clearance and the prespecified secondary objective was to compare 24 hours VAS pain; hospital stay, hematuria, protocol-defined postoperative sepsis, retreatment, and single-session stone-free status were assessed as additional exploratory outcomes.

## METHODOLOGY

Six months, single-center, open-label, parallel-group randomized controlled trial was conducted at the Department of Urology, Dr. Ziauddin Hospital, North Nazimabad, Karachi, from September 5, 2025 to March 4, 2026.

### Ethical approval:

Following Institutional Review Board approval, this single-center, open-label, parallel-group randomized controlled trial (NCT07261995) was conducted in the Department of Urology at Dr. Ziauddin Hospital, North Nazimabad, Karachi (IRB No. 10800725WAURO; September 5, 2025). The study duration was six months, from 5 September 2025 to 4 March 2026. Written informed consent was obtained before enrollment, and participant confidentiality was maintained throughout the study.

Eligible patients were enrolled by non-probability consecutive sampling. The sample size calculation was performed in OpenEpi version 3.01 using a two-sample comparison of proportions with a two-sided alpha of 0.05 (95% confidence), 80% power, expected clearance rates of 60% with ESWL and 85% with RIRS, and an absolute difference of 25%.^12^ The calculated requirement was 48.88 participants per group and was rounded to 49 per group (total n=98). To obtain the required randomized sample, 110 patients were assessed for eligibility.

At enrollment, baseline variables were recorded on a structured proforma, including age, gender, body mass index, and preoperative serum creatinine. Non-contrast computed tomography of kidneys, ureters, and bladder (CT-KUB) documented maximal stone diameter in millimeters, stone location (upper, middle, lower calyx, or renal pelvis), and hydronephrosis.

Patients aged 18-65 years with a single renal calculus measuring 1-2 cm on baseline non-contrast CT-KUB were included; all enrolled participants underwent CT-KUB before treatment. Exclusion criteria were multiple or bilateral renal stones; horseshoe kidney, ureteropelvic junction obstruction, or calyceal diverticulum; active urinary tract infection; pregnancy; a bleeding disorder or anticoagulation that could not be safely stopped; solitary kidney; estimated glomerular filtration rate <30 mL/min/1.73 m²; or body mass index >40 kg/m².

After baseline assessment, participants were randomized 1:1 by lottery. An independent research assistant prepared 98 identical opaque folded allocation slips, 49 labelled ESWL and 49 RIRS, which were mixed in a concealed container and opened only after enrollment. No blocking or stratification was used. The independent research assistant prepared and concealed the allocation slips; the study investigator enrolled participants and opened the selected slip after enrollment, and the treating urology team delivered the assigned intervention. ESWL was performed as a day-care procedure under sedation using the departmental fluoroscopy-guided lithotripter. Shock delivery began at 60 impulses/min and was increased to 100 impulses/min, with a maximum of 3000 shocks per session. When clinically significant residual fragments persisted, repeat ESWL was allowed at seven to 14 days intervals for a maximum of three sessions. RIRS was performed under general anesthesia after placement of a 5-Fr double-J stent two to three weeks before the definitive procedure. A flexible ureteroscope was advanced through a ureteral access sheath, and stone disintegration was performed with a Holmium: YAG laser at 0.5-1.2 J and 20-40 Hz using dusting or fragmentation as appropriate. A double-J stent was left after RIRS and removed after three weeks. Per institutional protocol, active urinary infection was excluded before intervention, peri-procedural antibiotic prophylaxis and hydration advice were provided, and the same paracetamol-based postoperative analgesic regimen was used in both groups. Discharge required stable vital signs, controlled pain on oral analgesia, tolerance of oral intake, and satisfactory voiding without significant hematuria or obstruction. All procedures were performed or directly supervised by consultant urologists with at least 10 years of experience.

Baseline non-contrast CT-KUB was performed in all participants before treatment. Follow-up CT-KUB at 28 days was interpreted by a consultant radiologist blinded to treatment allocation. True stone-free status was defined as complete absence of detectable stone; stone clearance, the registered primary outcome, was defined as no visible stone or a clinically insignificant residual fragment measuring ≤4 mm. Hospital stay for the index treatment admission was counted from admission to discharge, with same-day discharge recorded as zero; RIRS pre-stenting and subsequent stent-removal encounters were not included. Gross hematuria within 24 hours was documented in the clinical record and supported by urine microscopy. Protocol-defined postoperative sepsis (SIRS-based) was the prespecified infection outcome assessed within 72 hours using temperature ≥38°C, heart rate >90/min, respiratory rate >20/min, and white blood cell count >12,000/mm³. Both groups received the same paracetamol-based postoperative analgesic protocol, and pain was measured at 24 hours using a 10-cm Visual Analogue Scale scored from 0 to 10; trained nursing staff recorded the score before additional rescue analgesia where feasible. Retreatment was defined as any additional urological intervention for the same stone because of residual fragments on the 28 days CT-KUB or earlier CT-confirmed symptomatic obstruction or pain. The registered secondary outcome was 24-hour VAS pain; hospital stay, hematuria, protocol-defined postoperative sepsis, retreatment, and single-session stone-free status were analyzed as additional exploratory outcomes. All 98 randomized participants received their allocated intervention, completed 28 days follow-up, and were included in the analysis. There were no crossovers, protocol deviations, exclusions after randomization, or missing primary-outcome data; therefore, no imputation was required.

### Statistical analysis:

Data were analyzed using Statistical Package for the Social Sciences version 26. Normality of continuous variables was assessed using the Shapiro-Wilk test. Continuous variables, including age, body mass index, serum creatinine, hemoglobin, total leucocyte count, stone size, Hounsfield units, ESWL operative time for the first treatment session only, RIRS operative time for the definitive procedure, and 24 hours VAS score, were assessed for distribution. Normally distributed continuous variables were summarized as mean ± standard deviation and compared using independent-samples t-tests. The 24 hours VAS score was non-normally distributed on Shapiro-Wilk testing, and was summarized as median (IQR) and compared using the Mann-Whitney U test. Categorical variables were summarized as frequencies and percentages and compared using the Pearson chi-square test or Fisher exact test, as appropriate. Hospital stay was analyzed as a categorical variable. Mean differences (MDs) with 95% confidence intervals (CIs) were reported for continuous effect estimates and risk ratios (RRs) with 95% CIs for binary outcomes. A two-sided p-value <0.05 was considered statistically significant.

## RESULTS

There were 98 patients in all, divided equally between the groups receiving extracorporeal shock wave lithotripsy (ESWL) (n = 49) and retrograde intrarenal surgery (RIRS) (n = 49). The two groups demonstrated comparable profiles across all measured baseline variables (Table-I).

**Table-I:** Baseline and Demographic Characteristics of Study Participants (N=98)

Variable	ESWL (n=49) n (%) or mean±SD	RIRS (n=49) n (%) or mean±SD	p-value
***Age (years)***			
Mean ± SD	41.47 ± 9.41	41.35 ± 8.99	0.948
***Gender***			
Male	27 (55.1%)	32 (65.3%)	0.302
Female	22 (44.9%)	17 (34.7%)	—
***Body Mass Index (BMI, kg/m²)* Mean ± SD**	27.33 ± 3.63	26.73 ± 3.94	0.441
***Preoperative Laboratory Parameters***			
Serum creatinine (mg/dL), Mean ± SD	1.086 ± 0.263	1.104 ± 0.261	0.729
Hemoglobin (g/dL), Mean ± SD	13.33 ± 1.435	13.51 ± 1.157	0.487
Total leucocyte count (×10³/mm³), Mean ± SD	8.14 ± 1.744	8.12 ± 1.576	0.952
***Stone Characteristics***			
Stone size (mm), Mean ± SD	14.90 ± 2.435	14.76 ± 2.521	0.776
Hounsfield units (HU), Mean ± SD	807.65 ± 239.91	858.84 ± 235.49	0.289
***Stone Laterality***			
Right	26 (53.1%)	24 (49.0%)	0.686
Left	23 (46.9%)	25 (51.0%)	—
***Stone Location***			
Lower calyx	21 (42.9%)	21 (42.9%)	0.294
Middle calyx	9 (18.4%)	10 (20.4%)	—
Renal pelvis	9 (18.4%)	14 (28.6%)	—
Upper calyx	10 (20.4%)	4 (8.2%)	—
***Hydronephrosis***			
Present	19 (38.8%)	18 (36.7%)	0.835
Absent	30 (61.2%)	31 (63.3%)	—

Body Mass Index (BMI), Hounsfield Unit (HU), Extracorporeal Shock Wave Lithotripsy (ESWL), Retrograde Intrarenal Surgery (RIRS), and Standard Deviation (SD).

In the ESWL arm, one session achieved treatment completion in 28 patients (57.1%), while two and three sessions were required in 12 (24.5%) and nine (18.4%) patients, respectively. Intraoperative complications were observed only with RIRS, including hematuria in six (12.2%) and mucosal injury in five (10.2%); no ureteral perforation occurred. All RIRS patients received a double-J ureteric stent as per operative protocol, whereas none in the ESWL group were stented (χ²=98.00, p<0.001). Compared with ESWL, RIRS had a longer mean operative time (68.55±17.11 vs 44.55±7.94 minutes; MD 24.00 minutes, 95% CI 18.62 to 29.38; p<0.001) (Table-II).

**Table-II:** Intraoperative Characteristics and Procedural Variables (N=98)

Variable	ESWL (n=49) n (%) or mean±SD	RIRS (n=49) n (%) or mean±SD	p-value	MD (95% CI)
***Operative Time*** mean ± SD (minutes)†	44.55 ± 7.94	68.55 ± 17.11	<0.001	24.00 (18.62 to 29.38)
***Intraoperative Complications***				
Hematuria	0 (0.0%)	6 (12.2%)	0.027‡	—
Mucosal injury	0 (0.0%)	5 (10.2%)	0.056‡	—
Ureteral trauma	0 (0.0%)	0 (0.0%)	N/A¦	—
Perforation	0 (0.0%)	0 (0.0%)	N/A¦	—

ESWL = Extracorporeal Shock Wave Lithotripsy; RIRS = Retrograde Intrarenal Surgery; DJ = Double-J; MD = Mean Difference; N/A = Not Applicable. *Six RIRS patients sustained intraoperative hematuria; five sustained mucosal injury; 38 had no intraoperative complication. †For ESWL, operative time represents the first treatment session only. The MD is RIRS minus ESWL; unequal variances were assumed (Welch t=8.908, df=67.762, p<0.001). ‡Fisher exact test was applied when expected cell counts were <5. ¦No statistical test was computed because the variable was constant (zero events in both groups). Shaded rows indicate statistically significant differences (p<0.05).

Stone clearance was achieved in 33 of 49 patients (67.3%) after ESWL and 42 of 49 (85.7%) after RIRS at 28 days (RR 1.27, 95% CI 1.02 to 1.60; p=0.032). Complete stone-free status after the first session was 53.1% with ESWL and 85.7% with RIRS (RR 1.62, 95% CI 1.21 to 2.15; p<0.001). Hospital stay differed significantly (p<0.001): 71.4% of ESWL patients were discharged the same day and 28.6% on day one, whereas RIRS patients were discharged on day one (18.4%), day two (38.8%), day three (38.8%), or day four or later (4.1%). Median 24 hours VAS pain was 4 (IQR 3-4) after ESWL and 3 (IQR 2-4) after RIRS (Mann-Whitney U=685.5, p<0.001). Retreatment was required in 30.6% versus 10.2% (RR 0.33, 95% CI 0.13 to 0.85; p=0.012). Gross hematuria and protocol-defined postoperative sepsis were comparable between groups (Table-III). Steinstrasse occurred only after ESWL (10.2% vs 0%; Fisher exact p=0.056).

**Table-III:** Primary, Registered Secondary, and Additional Clinical Outcomes (N=98)

Outcome Variable	ESWL (n=49) n (%) or median (IQR)	RIRS (n=49) n (%) or median (IQR)	p-value	RR / MD (95% CI)
***Stone Clearance at 28 Days (Non-Contrast CT-KUB)***				
Stone clearance (no visible stone or residual fragment ≤4 mm), n (%)	33 (67.3%)	42 (85.7%)	0.032	1.27 (1.02 to 1.60)
Stone-free after first session, n (%)	26 (53.1%)	42 (85.7%)	<0.001	1.62 (1.21 to 2.15)
***Postoperative Pain Assessment***				
Pain VAS at 24 hours, Median (IQR)	4 (3-4)	3 (2-4)	<0.001	-0.96 (-1.42 to -0.49)
***Postoperative Hematuria (within 24 hours)***				
Gross hematuria, n (%)	6 (12.2%)	8 (16.3%)	0.564	1.33 (0.50 to 3.56)
***Protocol-defined postoperative sepsis (within 72 hours)***				
Protocol-defined postoperative sepsis, n (%)	3 (6.1%)	5 (10.2%)	0.461	1.67 (0.42 to 6.60)
***Retreatment***				
Retreatment required, n (%)	15 (30.6%)	5 (10.2%)	0.012	0.33 (0.13 to 0.85)

***ESWL =** Extracorporeal Shock Wave Lithotripsy; **RIRS =** Retrograde Intrarenal Surgery; **VAS =** Visual Analogue Scale; **CT-KUB =** Computed Tomography of Kidneys, Ureters and Bladder **IQR =** Interquartile Range; **RR =** Risk Ratio; **MD =** Mean Difference.*

Stone clearance was assessed by a consultant radiologist blinded to treatment allocation on non-contrast CT-KUB at 28 days using the registered ≤4 mm definition. The 24 hours VAS score was non-normally distributed on Shapiro-Wilk testing (ESWL p=0.004; RIRS p=0.005) and is presented as median (IQR); groups were compared using Mann-Whitney U (U=685.5, p<0.001). Effect estimates compare RIRS with ESWL: RRs are reported for binary outcomes, and the VAS MD (RIRS minus ESWL) is shown with its 95% CI. Hospital stay was analyzed as a categorical variable using Pearson chi-square across five ordinal categories. Retreatment was defined as any subsequent urological intervention for the same stone episode. Steinstrasse occurred only after ESWL and was compared using Fisher exact test (p=0.056). Statistically significant differences are indicated by shaded rows (p<0.05).

## DISCUSSION

The present trial showed higher 28 days stone clearance and single-session stone-free status with RIRS, with lower 24 hours pain and retreatment, whereas ESWL had shorter operative time and hospital stay; postoperative hematuria and protocol-defined postoperative sepsis were comparable. When treating 1-2 cm renal stones, retrograde intrarenal surgery was found to be more effective than extracorporeal shock wave lithotripsy, supporting the above hypothesis of more definitive stone control at this stone size. Conversely, extracorporeal shock wave lithotripsy had a distinct benefit in hospital stay, which indicates its day care profile. This observation is in line with the rest of the literature on stones in the range of one to two cm, where pooled evidence has indicated an improvement in stone-free rate with RIRS and a significantly increased rate of retreatment post-ESWL with no significant difference in overall complication grades.^13^ A recent review also concluded that RIRS achieves better clearance than ESWL for stones smaller than 2-cm, while ESWL more often requires repeat treatment.^14^retrograde intrarenal surgery (RIRS Similar findings were reported in a randomized trial of renal pelvis stones, in which short-term stone-free outcomes favored RIRS over ESWL.^15^ Comparable superiority of RIRS has also been observed in randomized and observational studies of lower-pole and mixed renal stones, particularly for stones approaching the upper end of this size range.^9^,^16^ Retreatment carries practical consequences because repeated sessions extend symptoms, follow-up, and radiological assessment despite extracorporeal shock wave lithotripsy being non-invasive. Studies focusing on lower pole stones have likewise reported higher ancillary procedures after extracorporeal shock wave lithotripsy, suggesting that fragmentation may not translate into timely clearance in all cases in practice.^9^,^17^

The shorter hospital stay seen with ESWL is also in keeping with previous literature and reflects its usual day-care or short-stay profile. Even in cases where RIRS produced better clearance, this benefit has been documented in both primary renal stone treatment and the management of residual fragments following percutaneous nephrolithotomy.^9^,^18^,^19^ Operative time was likewise shorter with ESWL, which is expected given the greater technical steps involved in RIRS.^17^ The ESWL group had a higher median 24 hours VAS pain score than the RIRS group. Postoperative pain results vary across studies; higher early pain after SWL has been described, particularly in lower calyceal stones.^18^ In contrast, stent-related discomfort may adversely affect early symptom scores after RIRS in some series.^16^

Postoperative hematuria and postoperative sepsis did not differ significantly between the two modalities, which is consistent with pooled evidence showing no major difference in overall complication burden between ESWL and RIRS.^20^ However, modality-specific adverse events remain relevant in clinical decision-making. Steinstrasse occurred only after ESWL in the present series, and this pattern has been described previously in comparative studies where it contributed to delayed clearance and the need for secondary intervention.^19^ A similar association between ESWL and steinstrasse has also been reported in lower calyceal stones.^17^,^19^ Intraoperative events were only observed in the retrograde intrarenal surgery arm due to endoscopic manipulation and did not translate into a higher burden of major postoperative morbidity, supporting an overall comparable short-term safety profile when patients are appropriately selected.

The current comparison provides clinically useful local evidence by considering CT-confirmed clearance, retreatment, index-admission profile, early pain, and short-term complications rather than stone-free status alone. Interpretation should remain within the study population and short follow-up period.

### Limitations

This single-center trial had a modest sample size and 28 days follow-up, limiting assessment of uncommon adverse events, longer-term residual-fragment passage, recurrence, and delayed complications. Participants and clinical staff could not be blinded because the procedures were readily distinguishable, although CT-KUB was interpreted by a blinded consultant radiologist. Restriction to adults with a single 1-2 cm renal stone and exclusion of complex, bilateral, solitary-kidney, severe chronic kidney disease, and morbidly obese cases may limit generalizability.

## CONCLUSION

Among adults with single renal stones measuring one to two centimeters, RIRS reported a comparatively higher stone clearance rate at the 28th day and greater single-session stone-free rates than ESWL. Postoperative pain and the need for retreatment were lower after retrograde intrarenal surgery. Extracorporeal shock wave lithotripsy offered shorter hospitalization, with most patients discharged the same day. Rates of complications were comparable between these surgical modalities. These findings support retrograde intrarenal surgery as the more favorable option when operative resources are available and may reduce procedural repetitions.

### Authors Contribution:

**MWA:** conception and design, data acquisition, manuscript drafting, critical revision

**AJ:** Contributing to data analysis, manuscript drafting, critical revision.

**SA:** Contributing to data acquisition and interpretation, manuscript drafting, critical revision

**AGY:** Contributing to study supervision, data analysis, critical revision.

All authors have read and approved the final version and are also accountable for the integrity of the study.
